# Effect of chitosan and bioactive glass nanoparticles on the push-out bond strength of epoxy resin-based sealer: an in-vitro study

**DOI:** 10.1186/s12903-026-10109-8

**Published:** 2026-11-10

**Authors:** Mohammad Hasan Kotainy, Mohamed A. Gomaa, Mohamed M. Ibrahim

**Affiliations:** Department of Endodontics, Faculty of Dentistry, Mansoura University, Mansoura, 35516 Egypt

**Keywords:** AH plus, Obturation, TotalFill BC Sealer, Universal testing machine

## Abstract

**Background:**

This study evaluated the effect of incorporating chitosan nanoparticles (CHSNPs) and/or bioactive glass nanoparticles (BAGNPs) into AH Plus (AHP) on its push-out bond strength (PBS) after 7 and 90 days, with unmodified AHP as the reference formulation and TotalFill BC Sealer (TF) as an additional comparator.

**Methods:**

A total of 120 straight palatal roots of extracted maxillary molars were prepared to size X4 (40/.06). The specimens were divided into five groups (n = 24 each): AHP, AHP + 10 wt% CHSNPs, AHP + 10 wt% BAGNPs, AHP + 10 wt% CHSNPs + 10 wt% BAGNPs, and TF. Each group was divided into 7- and 90-day storage subgroups (n = 12 each). After cold lateral compaction and storage, PBS was determined in a middle-third slice from each root using a universal testing machine. Data were analyzed using two-way ANOVA and the chi-square test, with the significance level set at *P* < 0.05.

**Results:**

Sealer formulation and its interaction with storage period significantly affected PBS (*P* < 0.05), whereas the main effect of storage period was not significant (*P* > 0.05). At 7 days, all nanoparticle-modified AHP formulations exhibited higher PBS than unmodified AHP and TF (*P* < 0.05). After 90 days, AHP and AHP + CHSNPs + BAGNPs exhibited higher PBS than AHP + CHSNPs, and all AHP-based formulations exhibited higher PBS than TF (*P* < 0.05). From 7 to 90 days**,** PBS increased significantly in unmodified AHP and decreased significantly in AHP + CHSNPs (*P* < 0.05), whereas no significant storage-related changes were detected in the remaining formulations (*P* > 0.05).

**Conclusions:**

Under the study conditions, CHSNPs and/or BAGNPs enhanced the early PBS of AHP, but their effects after prolonged storage were formulation-dependent. The early benefit was not maintained with CHSNPs alone, whereas BAGNP-containing formulations showed PBS comparable to unmodified AHP after 90 days. Nanoparticle-modified AHP formulations exhibited higher PBS than TF at both storage periods.

## Background

A three-dimensional fluid-tight seal throughout the entire root canal is essential for successful root canal therapy. Although gutta-percha (GP) is the primary material used for root canal filling, it cannot adhere directly to root canal dentin. Therefore, a root canal sealer is applied between the GP core and dentin to fill interfacial spaces, improve adaptation, and maintain long-term sealing of the root canal filling [1].

Strong adhesion helps preserve the sealer–dentin interface during subsequent procedures, particularly post-space preparation and final restoration. Higher bond strength lowers the risk of filling dislodgement and helps maintain root canal filling stability over the long term. Therefore, the push-out bond strength (PBS) test is commonly used to assess the adhesion of root canal sealers to dentin [2].

Epoxy resin-based sealers (ERBSs) and calcium silicate-based sealers (CSBSs) are widely used in endodontic clinical practice. AH Plus (AHP; Dentsply DeTrey GmbH, Konstanz, Germany) is widely recognized as the reference resin-based sealer because of its favorable physicochemical properties, such as optimal flowability, lower solubility, and dimensional stability [3, 4]. Despite these advantages, ERBSs have limited antibacterial activity and lack intrinsic bioactivity [4, 5].

TotalFill BC Sealer (TF; FKG Dentaire SA, La Chaux-de-Fonds, Switzerland) is a premixed CSBS composed primarily of calcium silicate compounds and radiopacifying agents. Owing to their inherent bioactivity, CSBSs have gained considerable attention because they release calcium ions and promote hydroxyapatite deposition, which may support periapical tissue healing. They also exhibit excellent biocompatibility and antibacterial activity. Nevertheless, once the material has set, its removal is difficult and may complicate retreatment procedures [6–8].

Nanotechnology is now widely used in both medical and dental fields because of the advantages it provides over conventional materials. Particles are classified as nanomaterials when at least one dimension is smaller than 100 nm. Their nanoscale size, high surface-area-to-volume ratio, and unique physicochemical characteristics can improve the physicochemical, mechanical, and biological properties of materials [9, 10]. Recently, incorporating bioactive and antimicrobial nanoparticles into ERBSs has attracted considerable interest as an effective strategy to enhance their antibacterial efficacy and bioactive potential [11–13].

Chitosan nanoparticles (CHSNPs) are naturally derived polysaccharide nanomaterials that exhibit favorable biological, physicochemical, and antimicrobial properties, supporting their use in endodontic applications [1, 11]. Incorporating CHSNPs into root canal sealers has been shown to enhance antibacterial activity while reducing cytotoxicity compared with the unmodified formulation [11]. Moreover, CHSNP incorporation resulted in significantly greater sealer penetration than that observed with the corresponding unmodified sealers [14].

Bioactive glass nanoparticles (BAGNPs) are primarily composed of varying proportions of silicon dioxide (silica), calcium oxide, sodium oxide, and phosphorus pentoxide and have been extensively applied in dentistry because of their capacity to promote hydroxyapatite formation in biological environments. They have attracted considerable attention as bioactive biomaterials owing to their excellent biocompatibility and their ability to promote mineralization and tissue repair [12]. Incorporating BAGNPs into epoxy resin-based sealers (ERBSs) has been shown to enhance their bioactivity compared with the corresponding unmodified sealers [12, 13].

Limited evidence is available regarding the effect of incorporating CHSNPs and/or BAGNPs into ERBSs on bonding to root canal dentin after different storage periods [1, 13]. Therefore, this study aimed to evaluate the effect of incorporating CHSNPs and/or BAGNPs into AHP on its PBS after 7- and 90-day storage periods, with unmodified AHP serving as the reference formulation and TF included as an additional comparator. The null hypotheses were that (1) sealer formulation would have no significant effect on PBS, (2) storage period would not significantly affect PBS, and (3) there would be no interaction between sealer formulation and storage period.

## Methods

### Ethical considerations and study design

The study protocol was approved by the Research Ethical Committee, Faculty of Dentistry, Mansoura University (Protocol No. MS.25.06.39).

**Fig. 1:**
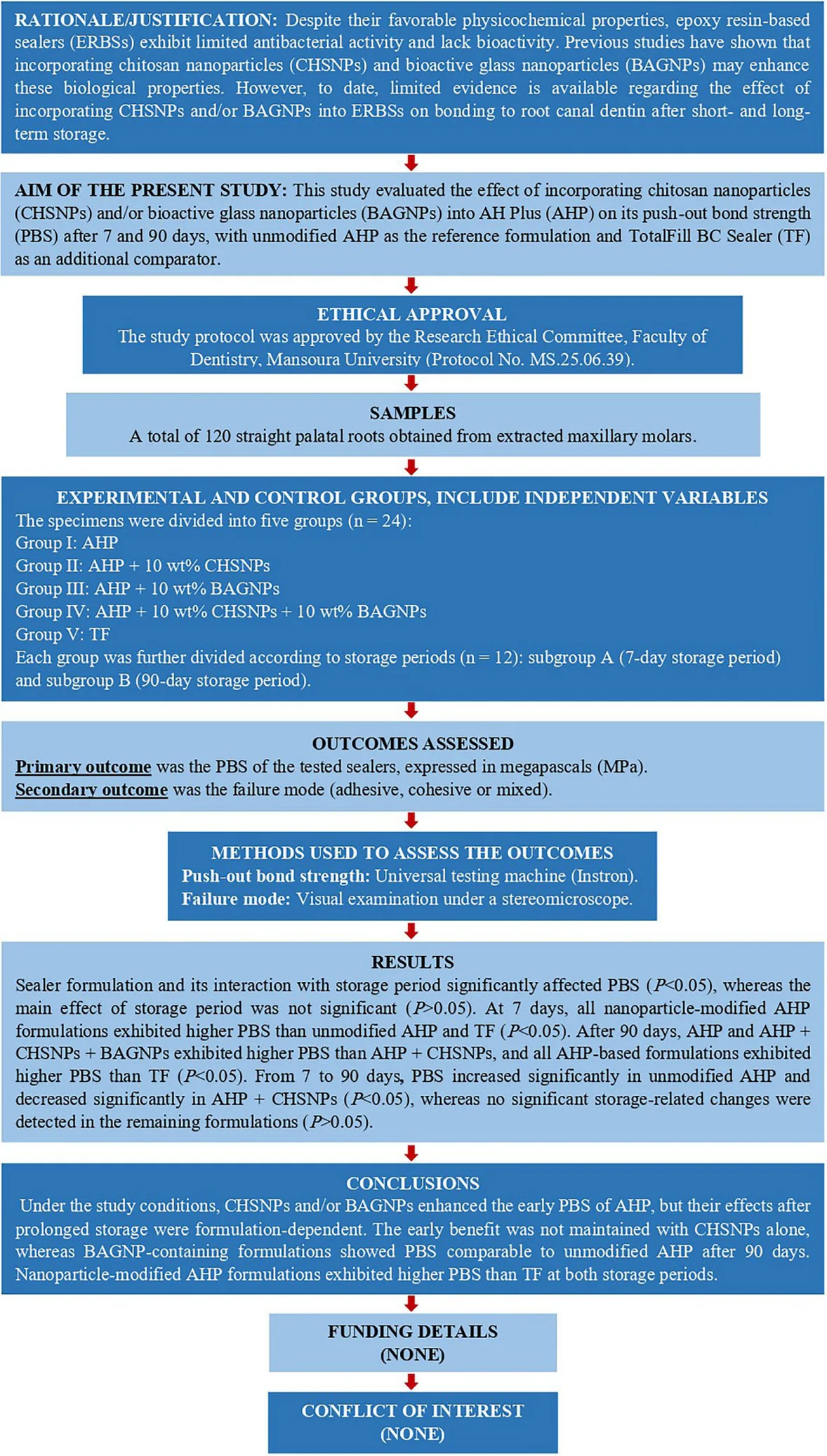
PRILE flowchart of the study design

**Fig. 2:**
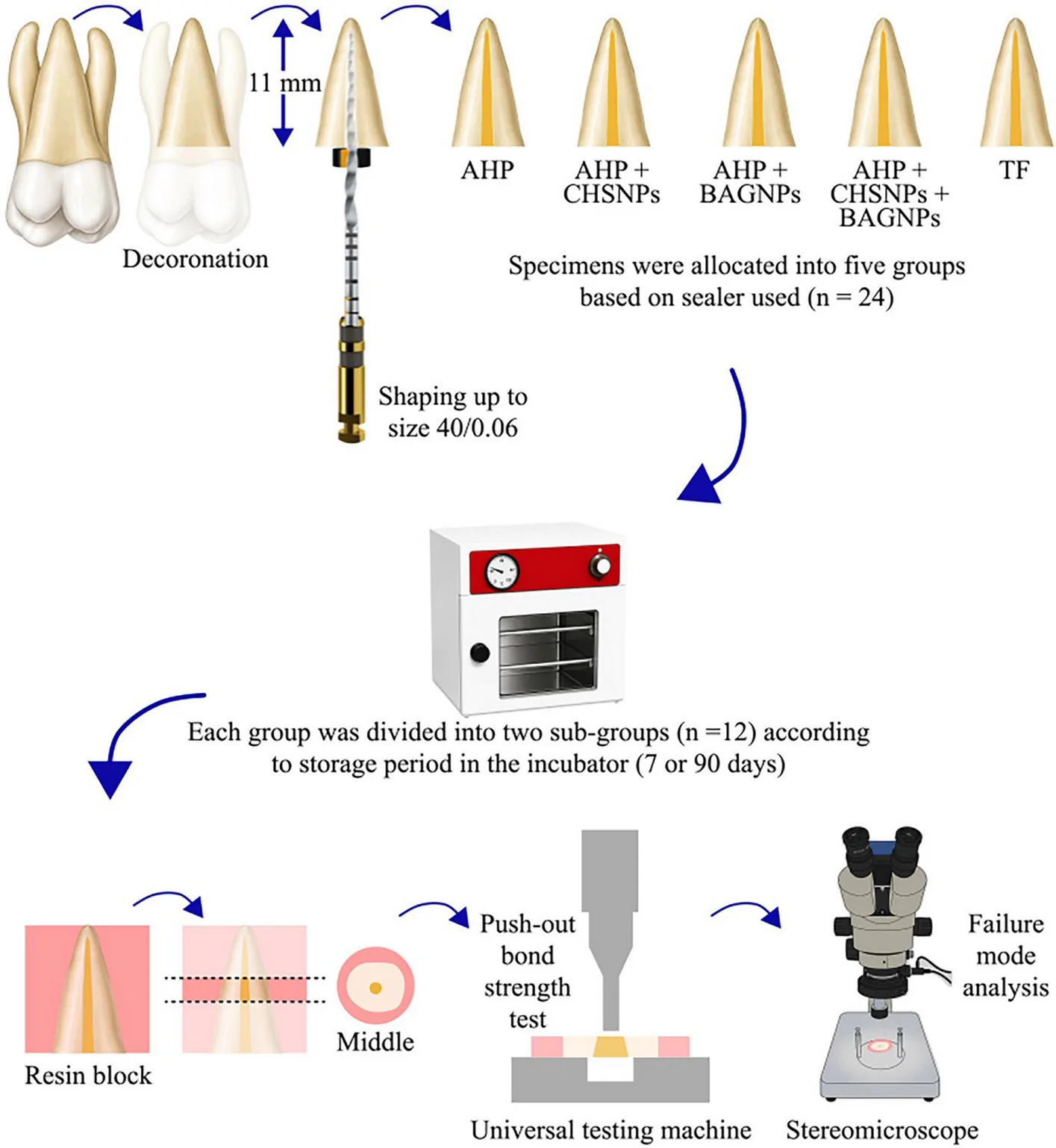
Schematic representation of the research methodology

The Preferred Reporting Items for Laboratory Studies in Endodontology (PRILE) 2021 guidelines were followed in this study (Fig. 1) [15]. The experimental workflow is schematically illustrated in (Fig. 2).

### Sample size calculation

The sample size was calculated using G*Power software (version 3.1; Heinrich Heine University, Düsseldorf, Germany). Based on previous similar studies [1, 2], a large effect size (Cohen’s f = 0.40) was assumed, with a significance level (α) of 0.05 and a statistical power of 94%, resulting in a minimum required sample size of 117 specimens. To ensure balanced allocation across the experimental subgroups, a total of 120 specimens (12 specimens per subgroup) were included in the study.

### Synthesis of nanoparticles

#### Chitosan Nanoparticles (CHSNPs)

CHSNPs were synthesized by ionic gelation through the interaction between chitosan and sodium tripolyphosphate (STPP). Chitosan (Oxford Lab Fine Chem LLP, Mumbai, India) was dissolved in 2.0% (v/v) acetic acid and stirred at 150 rpm at room temperature. An aqueous STPP solution (0.75 mg/mL) was then added slowly under continuous stirring, and the suspension was stirred at 500 rpm for 2 h to obtain a homogeneous suspension. The suspension was freeze-dried (Alpha 1–2 LDplus; Martin Christ, Germany) to obtain dry CHSNP powder [16, 17].

#### Bioactive Glass Nanoparticles (BAGNPs)

BAGNPs were synthesized using a modified sol–gel (Stöber) method with a 45S5 composition. Tetraethyl orthosilicate (TEOS; Sigma-Aldrich, USA) was dissolved in ethanol, followed by the addition of deionized water (water: TEOS = 4:1) under continuous stirring. Triethyl phosphate (TEP; Loba Chemie, India) and calcium nitrate (Loba Chemie, India) were subsequently added at 30-min intervals, followed by stirring of the mixture for a further 1 h. The obtained gel was heat-treated at 80 °C, dried at 140 °C, and calcined at 650 °C to obtain BAGNPs [18, 19].

### Characterization of nanoparticles

Transmission electron microscopy (TEM; JEM-2100, JEOL Ltd., Tokyo, Japan) was employed to examine nanoparticle morphology. The microscope was operated at an accelerating voltage of 200 kV. A Zetasizer Nano ZS (Malvern Panalytical, Malvern, UK) was employed to determine the particle size distribution and zeta potential of the nanoparticles.

### Teeth selection and preparation

One hundred and twenty intact maxillary molars extracted for periodontal disease were included in the study. The teeth were disinfected with 5.25% sodium hypochlorite (NaOCl; Prime Dental Products Pvt. Ltd., India) and subsequently cleaned using a P5 Newtron XS ultrasonic scaler (Satelec Acteon, Mérignac, France). The specimens were examined under a dental operating microscope (M320, Leica Microsystems, Wetzlar, Germany), and standardized mesiodistal and buccolingual radiographs were obtained using the paralleling technique with a digital X-ray sensor (NanoPix, Eighteeth, Changzhou, China). Teeth exhibiting cracks or internal or external root resorption were excluded, and the radiographs were used to confirm the root canal anatomy.

Inclusion criteria were palatal roots with a single canal, fully developed apices with patent foramina, absence of canal calcification, and absence of internal or external resorption. Exclusion criteria were immature apices, previously root canal–treated teeth, root caries, and roots with cracks or resorption.

The specimens were stored in 0.9% saline (Ateco Pharma, Egypt) at room temperature and used within two months of extraction. Using a diamond disc under continuous water cooling, the palatal roots were decoronated to a standardized length of 11 mm from the root apex.

### Nanoparticle incorporation into root canal sealers

AHP was modified by incorporating CHSNPs or BAGNPs at a final concentration of 10 wt%, corresponding to a nanoparticle-to-unmodified sealer weight ratio of 1:9. For the combination group, CHSNPs and BAGNPs were each incorporated at a final concentration of 10 wt%, resulting in a total nanoparticle loading of 20 wt%. The nanoparticles were incorporated into the base paste before mixing with the catalyst paste. All materials were accurately weighed using an analytical balance (accuracy: 0.0001 g) to standardize the incorporation of nanoparticles.

### Root canal preparation

The working length (WL) was determined with a size 10 K-file (Mani, Tochigi, Japan) positioned 1 mm short of the apical foramen and confirmed radiographically using a size 15 K-file. Root canal preparation was performed using the ProTaper Next rotary system (Dentsply Sirona, Charlotte, NC, USA) driven by an endodontic motor (E-Connect Pro, Eighteeth, Changzhou, China) to file X4 (40/0.06). Instrumentation was performed at 300 rpm and a torque of 2 Ncm using a gentle brushing motion [20]. Apical patency was maintained throughout instrumentation with a size 10 K-file. Following each instrument, the canal was irrigated with 3 mL of 5.25% (NaOCl) (Prime Dental Products Pvt. Ltd., India) using a side-vented 30-gauge irrigation needle (Irriflex, PD Dental, Vevey, Switzerland) positioned 2 mm short of the WL. Final irrigation consisted of 5 mL of distilled water, followed by 5 mL of 17% EDTA (Prevest, India), which was ultrasonically activated for 1 min using an IrriSafe tip (Satelec Acteon, Mérignac, France) driven by a P5 Newtron XS ultrasonic unit at a power setting of 6. A final rinse was performed with 5 mL of distilled water.

### Grouping of the specimens

The specimens were allocated into five groups (n = 24) according to the sealer used for obturation. Group I: AHP; Group II: AHP modified with 10 wt% CHSNPs; Group III: AHP modified with 10 wt% BAGNPs; Group IV: AHP modified with 10 wt% CHSNPs and 10 wt% BAGNPs (20 wt% total nanoparticle loading); and Group V: TF. Each group was further divided into two subgroups (n = 12) according to the storage period (7 or 90 days).

### Obturation of the root canals

For Groups (I–IV), size 40 paper points were used to dry the canals, whereas the canals in Group (V) were dried for 5 s with a low-suction vacuum adapter tip and for 1 s with a size 40 paper point [21]. Obturation was performed by the cold lateral compaction technique using a size 40/0.04 master gutta-percha cone (Meta Biomed, Cheongju, Korea), fitted to the working length with adequate tug-back, followed by size 25/0.02 accessory cones. A heated tip of the EQ-V Pack (Meta Biomed, Cheongju, Korea), set at 180 °C, was used to remove the excess gutta-percha, and the remaining gutta-percha was vertically compacted with a hand plugger (Fanta, Shanghai, China). Cavit (3M ESPE, Seefeld, Germany) was used to seal the coronal opening, and obturation quality was verified radiographically using a digital intraoral sensor (Woodpecker, Guilin, China). The roots were wrapped in gauze soaked in phosphate-containing simulated tissue fluid (Gibco, Life Technologies, Carlsbad, CA, USA) and stored at 37 °C under 100% humidity for either 7 days to allow complete sealer setting or 90 days to evaluate the effect of prolonged storage. The simulated tissue fluid was refreshed every 2 days.

### Preparation of specimens for the push-out bond strength test

The roots were vertically embedded in self-curing acrylic resin using cylindrical plastic molds (10 × 11 mm). After complete polymerization, roots were cut perpendicular to the long axis using a precision water-cooled saw (IsoMet 4000, Buehler, USA) with a 0.6 mm diamond blade. Single root slice (2.0 ± 0.1 mm) was obtained from the middle third of each root at a level of 4.5–6.5 mm from the apex. Slice thickness was checked using a digital caliper.

### Push-out bond strength test

For PBS testing, each specimen was secured in a custom-made metal holder using sticky wax. A flat-ended stainless-steel plunger (0.5 mm in diameter) was used to apply a compressive force at a crosshead speed of 0.5 mm/min using a universal testing machine (Instron Corp., Massachusetts, USA). To ensure appropriate force application, the plunger was chosen to be within 60–85% of the smaller canal diameter (apical side) of each specimen to contact only the filling material during testing without touching the root dentin [22, 23]. The plunger was oriented perpendicular to the specimen. The maximum load needed to dislodge the filling material was recorded in Newtons (N) and used to calculate the PBS in megapascals (MPa) using the following equation [24]:

$$\mathrm{P}\mathrm{B}\mathrm{S}=\mathrm{F}/\mathrm{A}=\mathrm{F}/\left(\pi \times \left(\left(\mathrm{D}1+\mathrm{D}2\right)/2\right)\times \mathrm{h}\right)$$

where:

F = maximum load (N).

A = Loaded surface area (mm^2^).

π = Constant (≈ 3.14).

D1 = Larger canal diameter (coronal side).

D2 = Smaller canal diameter (apical side).

h = Root slice thickness (mm).

### Failure mode analysis

Failure patterns were evaluated at 40 × magnification using a stereomicroscope (SMZ745, Nikon Corp., Tokyo, Japan). Based on the amount of sealer remaining on the dentin surface, failures were classified as adhesive (complete detachment from the dentin, with no sealer remaining), cohesive (failure occurred within the sealer, with the sealer attached to the dentin), or mixed (combined cohesive and adhesive failure, leaving part of the sealer on the canal walls) [24].

### Statistical analysis

Statistical analysis was performed using GraphPad Prism 10.0 (GraphPad Software, San Diego, CA, USA). Data normality was evaluated using the Shapiro–Wilk test. PBS values were expressed as mean ± standard deviation (SD). Two-way analysis of variance (ANOVA) was used to evaluate the effects of sealer formulation, storage period, and their interaction on PBS, followed by Tukey’s post hoc test for multiple comparisons. Failure mode distribution was analyzed using the chi-square test. Statistical significance was set at *P* < 0.05.

## Results

### Nanoparticle characterization

**Fig. 3:**
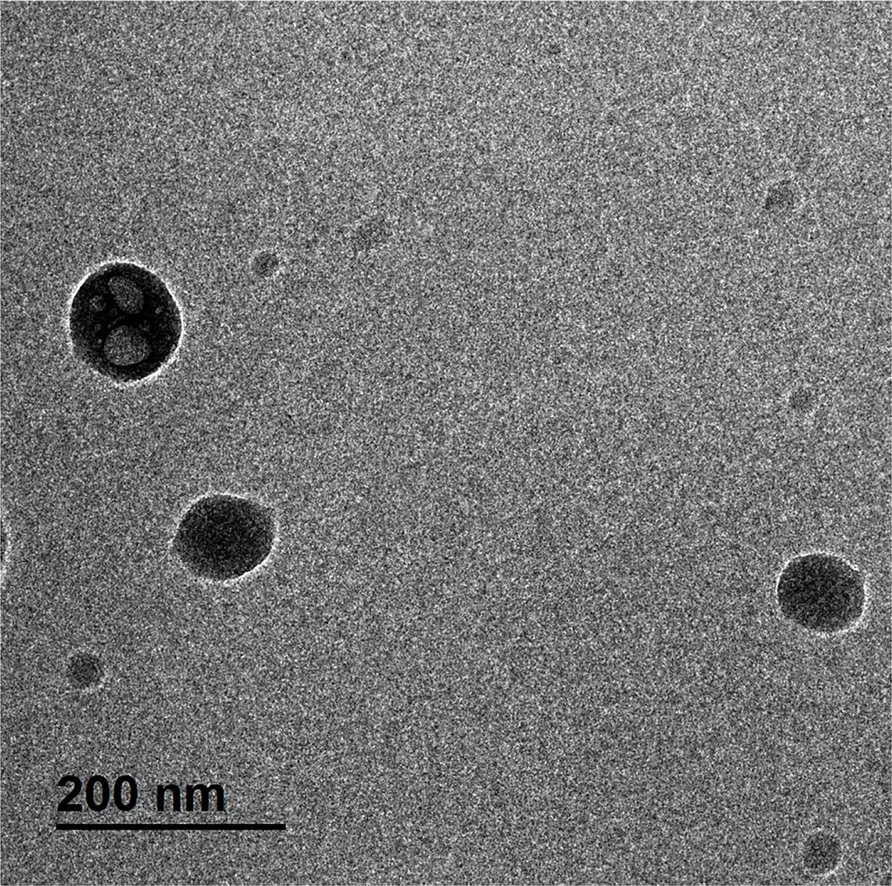
TEM analysis of CHSNPs

**Fig. 4:**
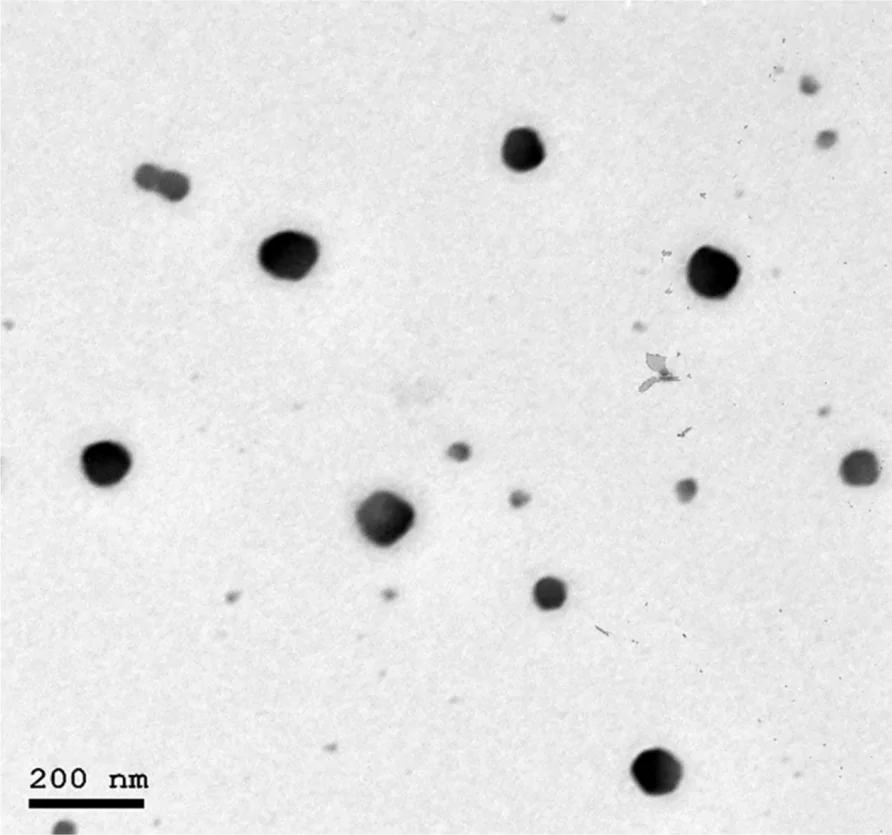
TEM analysis of BAGNPs

TEM micrographs showed that the synthesized CHSNPs and BAGNPs were predominantly spherical, well dispersed, and had particle sizes below 100 nm by TEM (Figs. 3 and 4). DLS analysis showed Z-average hydrodynamic diameters of 186.9 nm for CHSNPs and 154.9 nm for BAGNPs, with PDI values of 0.261 and 0.335, respectively. The zeta potential values were + 31.8 ± 4.9 mV for CHSNPs and − 21.9 ± 0.8 mV for BAGNPs.

### Push-out bond strength (PBS)

Two-way ANOVA showed significant effects of sealer formulation (P < 0.0001) and the interaction between sealer formulation and storage period (P < 0.0001), whereas the main effect of storage period was not significant (P = 0.1847) (Table 1).

**Table 1:** Results of the two-way ANOVA for sealer type, storage period, and their interaction on PBS

	**Sum of Squares**	**df**	**Mean Square**	**F**	***P*-value**	**Partial η**^**2**^
Sealer	204.5	4	51.13	30.89	< 0.0001^*^	0.529
Storage Period	2.949	1	2.949	1.782	0.1847	0.016
Sealer* Storage Period	127.9	4	31.96	19.31	< 0.0001^*^	0.413
Residuals	182.1	110	1.655			
Total	517.4	119				

*df* degrees of freedom, *F* Fisher’s F -statistic, *ƞ*_*p*_^*2*^ partial eta squared

^*^Statistically significant difference at *P* < 0.05

When data were pooled across storage periods, AHP + CHSNPs, AHP + BAGNPs, and AHP + CHSNPs + BAGNPs exhibited significantly higher PBS than unmodified AHP and TF (P < 0.05). Unmodified AHP also showed significantly higher PBS than TF (P < 0.05), while no significant differences were detected among the three nanoparticle-modified AHP formulations (P > 0.05) (Table 2).

**Table 2:** PBS pooled data means [MPa] among the studied groups, regardless of storage period

Group	Mean ± SD	*P*-value
AHP (n = 24)	5.686 ^b^ ± 2.1	< 0.0001*
AHP + CHSNPs (n = 24)	6.81 ^a^ ± 1.717	
AHP + BAGNPs (n = 24)	7.474 ^a^ ± 1.474	
AHP + CHSNPs + BAGNPs (n = 24)	7.478 ^a^ ± 1.376	
TF (n = 24)	4.037 ^c^ ± 1.475	

^*^Different lowercase superscript letters indicate significant difference at *P* < 0.05

Because a significant sealer formulation × storage period interaction was detected, comparisons were further examined within each storage period. After 7 days, all three nanoparticle-modified AHP formulations showed significantly higher PBS than unmodified AHP and TF (P < 0.05). No significant differences were observed among the nanoparticle-modified formulations or between unmodified AHP and TF (P > 0.05).

**Fig. 5:**
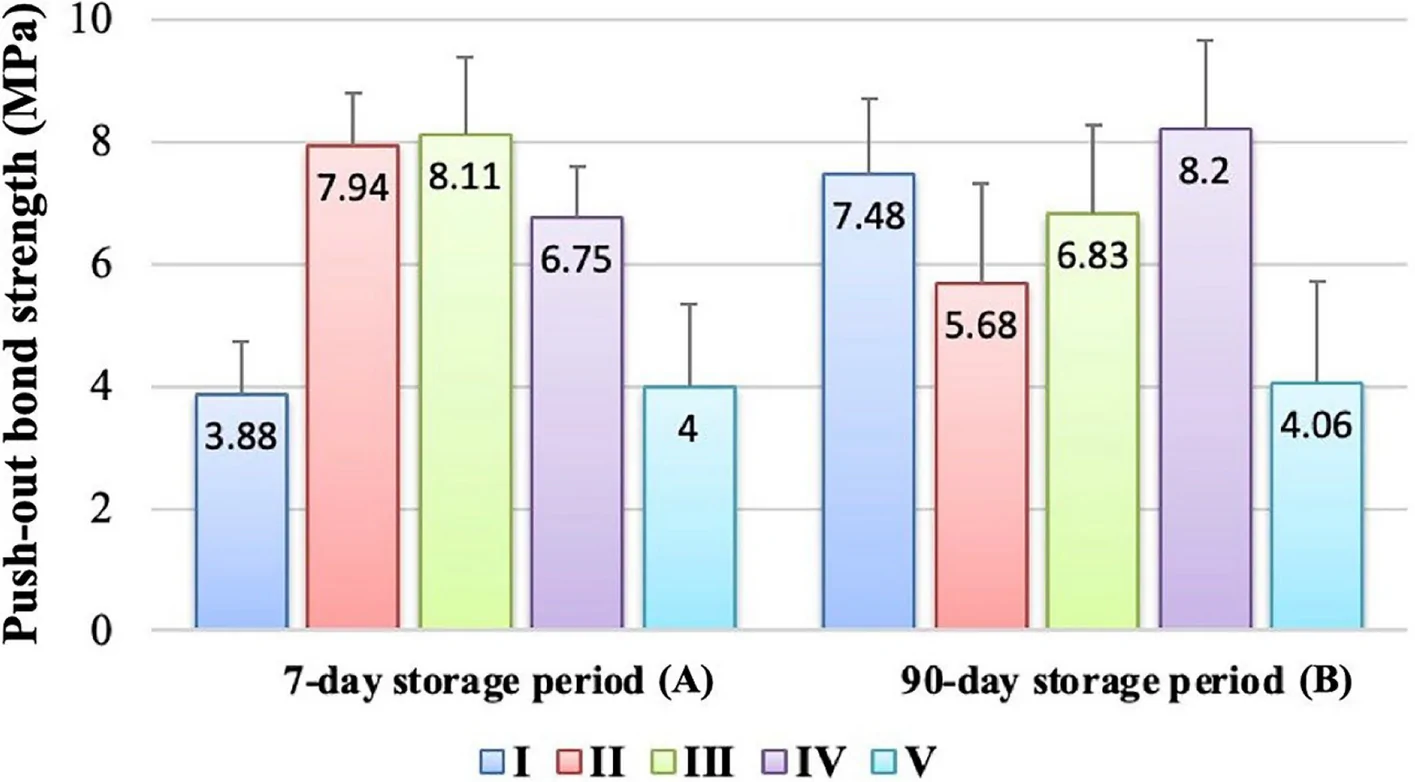
Bar chart showing mean PBS of the tested sealers after 7- and 90-day storage periods. I, AHP; II, AHP + CHSNPs; III, AHP + BAGNPs; IV, AHP + CHSNPs + BAGNPs; V, TF. Error bars represent standard deviations

After 90 days, unmodified AHP and AHP + CHSNPs + BAGNPs exhibited significantly higher PBS than AHP + CHSNPs (P < 0.05). No significant differences were detected among unmodified AHP, AHP + BAGNPs, and AHP + CHSNPs + BAGNPs, or between AHP + CHSNPs and AHP + BAGNPs (P > 0.05). All AHP-based formulations exhibited significantly higher PBS than TF (P < 0.05) (Fig. 5).

Within each formulation, unmodified AHP showed significantly higher PBS after 90 days than after 7 days, whereas AHP + CHSNPs showed significantly lower PBS after 90 days (P < 0.05). No significant storage-related differences were observed for AHP + BAGNPs, AHP + CHSNPs + BAGNPs, or TF (P > 0.05) (Fig. 5).

### Failure mode analysis

Failure mode distribution did not differ significantly among the tested subgroups (P > 0.05). Mixed failure was the most frequently observed pattern in all subgroups (Table 3).

**Table 3:** Distribution of failure modes after the PBS

	**7-Days**			**90-Days**		
	**Failure modes**			**Failure modes**		
	**Adhesive**	**cohesive**	**mixed**	**Adhesive**	**cohesive**	**mixed**
AHP	1	4	7	2	4	6
AHP + CHSNPs	1	4	7	1	3	8
AHP + BAGNPs	3	4	5	1	2	9
AHP + CHSNPs + BAGNPs	2	1	9	1	2	9
TF	1	5	6	1	4	7
	χ^2^ = 5.85 *P*-value = 0.6641			χ^2^ = 2.872 *P*-value = 0.9421		
	**χ**^**2**^** = 9.895** *P*-value = 0.9353					

χ^2^: chi-square statistic. No significant difference was observed when *P* > *0.05*

## Discussion

Endodontic sealers play a key role in achieving a durable fluid-tight seal, which is a primary objective of root canal treatment. Therefore, adequate bond strength to radicular dentin is considered a clinically important property, as it minimizes gap formation at the sealer–dentin interface and enhances the resistance of the root filling to dislodgement during functional loading or post space preparation, thereby supporting the long-term success of root canal therapy [23, 25].

Specimen standardization is essential for obtaining reliable and reproducible PBS measurements. Accordingly, straight palatal roots of maxillary molars with uniform lengths were instrumented using the ProTaper Next X4 (40/0.06) system to achieve comparable canal dimensions compatible with the universal testing machine plungers [26]. The ProTaper Next system was selected for its favorable shaping ability and preservation of the original canal anatomy [20]. In addition, specimens were prepared from the middle third of the obturated roots because its relatively uniform canal diameter improves standardization [27]. Previous evidence has demonstrated significant differences in PBS between AHP and bioceramic sealers at this root level [25]. Nevertheless, evaluating only the middle third does not account for possible regional variations in bond strength along the root canal, and future studies should compare PBS at different root levels, including the apical third.

To further minimize the influence of anatomical variability, the plunger size was selected to maintain a diameter corresponding to 60%–85% of the apical canal diameter (D2), thereby ensuring consistent stress distribution while accounting for differences in canal morphology [22, 23].

The moisture level within the root canal system plays an important role in sealer adhesion. Slightly moist canals have been associated with higher bond strength, particularly for hydrophilic sealers, whereas excessive moisture may lead to water accumulation at the sealer–dentin interface and reduce adhesion [21]. Excessive drying may likewise impair bonding by removing water from the dentinal tubules and limiting the penetration of hydrophilic sealers [28]. However, maintaining a consistent moisture level within the root canal remains a clinical challenge [29].

In the present study, different drying protocols were used according to the type of sealer. For AH Plus, a hydrophobic sealer that performs best under dry conditions, the canals were completely dried with paper points. For TF, a hydrophilic sealer with a moisture-activated setting reaction, the canals were dried using low suction with a vacuum adaptor for 5 s, followed by the use of a single paper point for 1 s. This approach was intended to preserve a controlled level of residual moisture, which is considered important for promoting effective adhesion of root canal sealers to dentin [21].

The cold lateral compaction technique was selected because it provides a relatively uniform sealer thickness at the core–dentin interface while ensuring adequate filling of the root canal system, thereby promoting effective adaptation of the sealer to the canal walls [30, 31]. Following obturation, all specimens were wrapped in gauze moistened with simulated tissue fluid and stored at 37 °C and 100% humidity. This storage protocol maintained a moist environment throughout the setting period. Simulated tissue fluid was used because its phosphate-containing composition has been reported to support the biomineralization and maturation of the CSBSs [32]. The 7-day storage period was selected to allow complete sealer setting before PBS evaluation, whereas the 90-day period was chosen to assess the effect of prolonged storage on sealer–dentin bonding. These time points are consistent with previous studies evaluating PBS over storage periods ranging from 1 week to 3 months [2, 33, 34].

Based on the two-way ANOVA findings, the null hypotheses regarding sealer formulation and the interaction between sealer formulation and storage period were rejected, whereas the null hypothesis regarding the main effect of storage period was not rejected. The significant formulation × storage period interaction indicated that the effect of storage on PBS depended on the sealer formulation; therefore, the findings were further interpreted according to storage period and formulation.

At the 7-day storage period, AHP + CHSNPs, AHP + BAGNPs, and AHP + CHSNPs + BAGNPs exhibited significantly higher PBS than both unmodified AHP and TF (*P* < 0.05), whereas no significant difference was detected between unmodified AHP and TF (*P* > 0.05). Because all nanoparticle-modified formulations were based on AHP, unmodified AHP served as the reference formulation for interpreting the effects of nanoparticle incorporation. The bonding of AHP to root canal dentin may be related to its physicochemical characteristics and the formation of covalent bonds between the sealer’s epoxide rings and the amino groups of dentinal collagen, which may contribute to its adhesion to dentin [22, 35].

In formulations containing CHSNPs, the higher early PBS values may be attributed to the nanoscale size and bioadhesive properties of chitosan nanoparticles, which may favor modified sealer penetration into dentinal tubules, improve adaptation at the sealer–dentin interface, and reduce interfacial gaps [14, 36]. The high surface area and reactive amino groups of CHSNPs may also facilitate hydrogen bonding, while chitosan nanoparticles have been shown to interact with and cross-link type I dentinal collagen, providing an additional potential mechanism for their interaction with the dentin substrate [1, 37].

Similarly, in the formulations containing BAGNPs, the higher early PBS values may be attributed to the bioactive properties of BAGNPs. Upon interaction with physiological fluids, BAGNPs undergo ion exchange, during which network-modifying ions, including Ca⁺^2^, are released from the glass in exchange with H⁺/H₃O⁺ from the surrounding medium, resulting in the formation of Si–OH groups on the glass surface. These groups subsequently condense into a silica-rich layer, providing nucleation sites for calcium-phosphate precipitation, which may subsequently mature toward hydroxyapatite [38, 39]. Early mineral deposition at the sealer–dentin interface may also contribute to the formation of tag-like structures, potentially improving mechanical retention [40].

The significant formulation × storage period interaction was further reflected in the different storage-related responses of the tested formulations. AHP showed significantly higher PBS after 90 days than after 7 days. This finding is consistent with previous studies reporting significant increases in the PBS of AHP from 7 to 90 days and over a shorter interval from 1 week to 1 month [33, 34].

In the AHP + CHSNPs group, PBS after 90-day storage was significantly lower than that after 7-day storage. The incorporation of hydrophilic CHSNPs into the relatively hydrophobic epoxy resin matrix of AHP may alter its water-related physicochemical behavior during prolonged aqueous storage. Recent evidence indicates that the physicochemical behavior of nanoparticle-modified epoxy resin-based sealers is influenced by nanoparticle type, loading, and dispersion, with particle agglomeration or structural defects potentially facilitating fluid penetration [10]. Moreover, ionically cross-linked chitosan/STPP nanoparticles have shown time-dependent changes in particle organization, including swelling and aggregation, during prolonged storage in phosphate buffer [41, 42]. Although these observations were obtained from free nanoparticles rather than particles embedded within a set epoxy sealer, they suggest that the CHSNP-containing phase may undergo time-dependent water-related changes during prolonged storage, potentially affecting the integrity of the modified resin matrix and the sealer–dentin interface.

In contrast, AHP + BAGNPs and AHP + CHSNPs + BAGNPs showed no statistically significant difference in PBS between 7 and 90 days. This storage-related behavior may be related to the time-dependent bioactivity of BAGNPs during aqueous exposure [13, 43]. A previous study on an epoxy resin sealer containing 10 wt% BAGNPs showed that calcium–phosphate-rich deposits formed after 2 weeks of immersion in simulated body fluid, became denser, and reached a Ca/P ratio within the hydroxyapatite range after 4 weeks, indicating progressive maturation of the initially precipitated calcium–phosphate phases [13]. Such time-dependent mineral maturation may have contributed to interfacial retention during prolonged storage.

Following prolonged storage (90 days), AHP and AHP + CHSNPs + BAGNPs exhibited significantly higher PBS than AHP + CHSNPs (*P* < 0.05). No significant differences were observed between AHP and AHP + BAGNPs or AHP + CHSNPs + BAGNPs (*P* > 0.05). In addition, AHP + CHSNPs did not differ significantly from AHP + BAGNPs (*P* > 0.05). All AHP-based formulations exhibited significantly higher PBS than TF at this storage period (*P* < 0.05).

TF showed no statistically significant storage-related change in PBS between 7 and 90 days. When data were considered across both storage periods, TF exhibited significantly lower overall PBS than AHP and the nanoparticle-modified AHP formulations. The comparatively lower overall PBS of TF may be related to its distinct bonding mechanism to root canal dentin. TF relies primarily on biomineralization and micromechanical interlocking; calcium ions released from the sealer may interact with phosphate ions and promote apatite formation at the sealer–dentin interface, while penetration into dentinal tubules may produce tag-like structures that contribute to mechanical retention [22]. Consistent with the present findings after prolonged storage, TF has been reported to exhibit significantly lower PBS than AHP after 8 weeks of incubation [22].

The dentin surface condition may also have influenced TF bonding. EDTA-induced smear-layer removal and alterations in dentin mineral composition have been associated with lower PBS of calcium silicate-based sealers [44]. These changes in dentin mineral content and surface chemistry may interfere with the calcium-phosphate interactions involved in apatite formation and development of the interfacial mineral infiltration zone [22, 44, 45].

Collectively, the distinct temporal responses of the tested formulations indicate that the effect of prolonged storage on PBS was formulation-dependent rather than uniform. Direct evidence regarding the long-term aging behavior of nanoparticle-modified epoxy resin-based sealers remains limited [10]. Further studies combining long-term physicochemical assessment with characterization of nanoparticle dispersion and the sealer–dentin interface are needed to clarify the underlying mechanisms.

Chi-square analysis of failure modes showed no statistically significant differences between the tested subgroups (*P* > 0.05). Mixed failure was the most frequently observed pattern in all subgroups, suggesting adequate bonding at the sealer–dentin interface, with failure extending through both the interface and the material itself. These findings are consistent with previous reports describing predominantly mixed failure patterns [2, 23].

As an in-vitro study, the experimental conditions could not fully reproduce the complex clinical environment. Before complete sealer setting, endodontically treated teeth may be subjected to functional occlusal loading, thermal fluctuations, and other intraoral challenges that could influence the sealer–dentin interface and bond strength. In addition, water-related physicochemical changes, nanoparticle dispersion within the set modified sealers, and chemical or morphological changes at the sealer–dentin interface during prolonged storage were not directly characterized. Failure mode analysis was performed using a stereomicroscope, which provides only limited visualization of the debonded interface. Scanning electron microscopy could provide a more detailed and objective evaluation of the sealer–dentin interface and failure patterns.

## Conclusions

Under the conditions of this study, CHSNPs and/or BAGNPs enhanced the early PBS of AHP, but their effects after prolonged storage were formulation-dependent. Unmodified AHP showed increased PBS over time, whereas the early benefit was not maintained with CHSNPs alone. BAGNP-containing formulations showed no significant storage-related changes and were comparable to unmodified AHP after 90 days. The nanoparticle-modified AHP formulations exhibited higher PBS than TF at both storage periods.

## Data Availability

This study has been extracted from MSc thesis conducted at Mansoura University. The data supporting the findings of this study are available from the corresponding author upon reasonable request.

## Abbreviations

AHP: AH Plus
BAGNPs: Bioactive glass nanoparticles
CHSNPs: Chitosan nanoparticles
CSBSs: Calcium silicate-based sealers
DLS: Dynamic light scattering
ERBSs: Epoxy resin-based sealers
GP: Gutta-percha
NaOCl: Sodium hypochlorite
PBS: Push-out bond strength
PDI: Polydispersity index
STPP: Sodium tripolyphosphate
TEM: Transmission electron microscopy
TF: TotalFill BC Sealer
